# The Prevalence of Compulsive Buying and Hoarding Behaviours in Emerging, Early, and Middle Adulthood: Multicentre Epidemiological Analysis of Non-clinical Chinese Samples

**DOI:** 10.3389/fpsyg.2021.568041

**Published:** 2021-12-09

**Authors:** Jiawen Ye, Simon Ching Lam, Heping He

**Affiliations:** ^1^Department of Applied Psychology, Lingnan University, Tuen Mun, Hong Kong SAR, China; ^2^School of Nursing, Tung Wah College, Ho Man Tin, Hong Kong SAR, China; ^3^College of Management, Shenzhen University, Shenzhen, China

**Keywords:** compulsive buying, compulsive hoarding, non-clinical Chinese sample, Richmond Compulsive Buying Scale, Hoarding Rating Scale, multi-center

## Abstract

Behavioural addictions, such as compulsive buying (CB) and hoarding, are increasingly recognised in the current psychiatric nosology, particularly in developed countries. The prevalence of these disorders may not be static but possibly altered across different age groups. However, studies on this area are rare, and only few have focused on Chinese population. This epidemiological study employs population-based cross-sectional design and collects data in two regions, i.e., Hong Kong and Mainland China. A self-reported questionnaire is constructed based on carefully validated Chinese versions of Richmond Compulsive Buying Scale and Hoarding Rating Scale. A total of 2,439 valid samples are collected and divided into three age groups, i.e., emerging, early and middle adulthood. CB and hoarding behaviours have displayed an inverted u-shaped pattern across the above age groups. In addition, the strength of correlation between the two compulsive behaviours is consistent and maintained across different age groups. These results suggest that the compulsive behaviours vary among different stages of adulthood in major cities in China. This newly discovered pattern of compulsive disorders in Chinese population is different from those in American and European populations.

## Introduction

Shopping is a common behaviour in modern life. People shop to buy their needs or relax and reward themselves. However, shopping can become a harmful and destructive behaviour to one’s life when it becomes extreme and unmanageable, which can lead to “compulsive buying” (CB). According to Ridgway et al. (2008), CB has two components, i.e., obsessive compulsive and impulsive control disorders (OCD and ICD, respectively). Compulsive buying is defined as “a consumer’s tendency to be preoccupied with buying that is revealed through repetitive buying and a lack of impulse control over buying” (Ridgway et al., 2008, p. 622).

CB behaviour (CBB) has attracted research attention since 1990s in developed societies because of its significant personal and social effects. Evidence has shown that CB may lead to large debts (58.3%), guilt (45.8%), inability to meet payments (41.7%), criticism from acquaintances (33.3%), legal and financial consequences (8.3%) and criminal legal problems (8%) (Christenson et al., 1994). In addition to financial and legal consequences, people with CBB may also suffer from an increasing level of anxiety and urge, which can only be relieved by acquiring a sense of completion after shopping (Black, 2007; Yi, 2013). Previous research has shown that compulsive buyers demonstrate anxiety disorders with obsessive thoughts and compulsive behaviours that lead to distress and disturbance to their everyday life (Billieux et al., 2008).

Although CBB has been proven to cause financial and psychological distress and can be considered as a type of behavioural addiction (Griffiths, 2005), CB is not listed as a separate category or a behavioural addiction in the American Psychiatric Association’s DSM-5 (American Psychiatric Association, 2013) or the World Health Association’s International Statistical Classification of Diseases and Related Health Problems, 10th revision (World Health Organization, 1992). One of the reasons is that CBB has no clear diagnostic criteria and sufficient data, and thus it merely overlaps with other mental disorders, such as OCDs, anxiety and/or depression (Weinstein et al., 2016). Moreover, existing research on CBB has used samples from industrialised countries in Europe and North America. Thus, evidence of CBB in Asian societies is little to none. Amongst existing studies on the prevalence of CBB that have used a population-based samples (excluding those that have used student samples because of their limited representativeness), the CB prevalence rates are found to be 5.8%, 6.9%, and 7.1% in 2,153 American, 2,350 German and 2,195 Spanish samples, respectively (Koran et al., 2006; Mueller et al., 2010; Otero-López and Villardefrancos, 2014). A meta-analysis research in 2016 has indicated that adult representative data with Chinese samples is scarce (Maraz et al., 2016). Amongst the few studies that have investigated university students, a study conducted in Mainland China using German Compulsive Buying Scale (Raab et al., 2005) has shown a CB prevalence rate of 6.7% (Li et al., 2014). Another research with Taiwanese undergraduate samples has suggested a prevalence rate of 29.8% with the adaptation of Faber and O’Guinn (1992) compulsive buying scale. The discrepancy between these two reported prevalence rates may be due to the different scales of CB used in these two studies. The scales used in the above studies are also not well validated in Chinese population. Therefore, a clear picture of CB in Chinese population awaits further investigation with a carefully validated Chinese version of CB scale. To investigate CBB in China, this epidemiological study adopts the Chinese version of Richmond Compulsive Buying Scale (RCBS), which has been recently validated by a group of researchers in Hong Kong (Lam et al., 2018). This study obtains sample data from Chinese residents of Hong Kong and Shenzhen, Mainland China, the two major Chinese cities with similar GDP (HK$2.85 trillion vs. HK$2.87 in 2018, Chan and Leung, 2019). Thus, the findings of this study are comparable to the past general population results found in western societies and significantly contribute to existing literature.

The tendency of CBB is found correlated to age. Specifically, young people seem to be prone to CBB. Research on the estimated prevalence of CBB in the United States has found that the identified compulsive buyers have a significantly lower mean age than other respondents (Koran et al., 2006). Dittmar’s (2005b) two empirical studies with student samples have provided unanimous findings that young consumers are engaged in more compulsive buying and showed concern that the younger generation’s CBB may negatively affect their finance and lead to great personal debts. Recruiting participants through various methods (e.g., university student research experience program, word of mouth, and media promotions, etc.), a study in Australia has studied adults aged 18–65 years and found that younger age predicted significantly severe excessive buying (Kyrios et al., 2020). One of the possible reasons for young adults’ vulnerability of CBB could be explained by the idea that CBB is an identity-seeking behaviour (Dittmar, 2005b). Younger adults are still in the stage of life to establish a sense of self and they may use shopping as an easy way to improve their self-image and move closer to their ideal self (Dittmar, 2005a). Most of the existing CBB studies have been conducted in western and developed societies. In Asia, research on CBB or excessive buying behaviours is still on its early stages and has been focused on adolescents and young adults. A comparison between Thailand and Chinese junior high school students has found that approximately 19% Chinese and 25% Thai adolescents are classified as compulsive buyers (Guo and Cai, 2011). A study conducted in Yantai, China has found that only 5.99% of the college student sample have CB tendency (Jiang and Shi, 2016). However, research on a more general population with people of different ages is scarce, and the prevalence of CBB may not be static but vary among different age groups as found in western societies, such as the United Kingdom, Spain and the United States (Dittmar, 2005b; Koran et al., 2006; Otero-López and Villardefrancos, 2014). To provide a comprehensive picture of the prevalence of CBB throughout the adulthood, a general population sample is adopted in this study to explore the relationship between age and CBB in China from emerging adulthood to middle adulthood, which includes the mostly studied young adults and seldom researched middle aged individuals. It is hypothesized that age is correlated to CBB, and younger individuals are more likely to demonstrate CBB (Hypothesis 1).

Studies on CB have consistently demonstrated that women are highly vulnerable to CBB. Women tend to consider shopping a positive leisure activity, whereas men perceive buying relatively negative and treat it as a task they have to finish (Campbell, 2000). Women usually score significantly higher than men on CB scales (Scherhorn et al., 1990; Dittmar, 2005b). Women have a greater level of susceptibility to CB in order to regulate emotions and moods (Davis et al., 2014). Another study findings suggested that female compulsive buyers may use excessive buying to cope with stress and negative feelings (Challet-Bouju et al., 2020). The pleasures and joy experienced in shopping exerted more strongly impacts women than men (Tarka et al., 2022). However, some studies with adolescent and university student sample in western countries have failed to find any gender differences (Roberts and Tanner, 2000; Lee and Workman, 2018). Another research with 410 German undergraduate student samples has even found that females have reported significantly lower levels of CBB than their male counterparts (Mueller et al., 2011). Therefore, the commonly assumed gender differences in CB are actually not that robust based on the existing evidence from western societies. On the other hand, in one of the few studies with Chinese sample, researchers have demonstrated that female college students in Hong Kong and Macau have scored significantly higher than their male counterparts in Edwards Compulsive Buying Scale (Ching et al., 2016). In consistent with the Western findings, a Chinese study on online compulsive buying among women also suggested that when experiencing high level of stress, some women may engage in online compulsive buying as a stress coping strategy, even though it is a negative one (Zheng et al., 2020). Hence, this study further explores the gender differences in a general Chinese sample and hypothesizes that women are more prone to CB than men (Hypothesis 2).

The relationship between compulsive hoarding (CH) and CB has attracted increasing research attention recently. Compulsive hoarding behaviours are described as obtaining a large number of possessions but failing to discard, which leads to substantial clutter that greatly affects the living space of an individual and his/her normal functioning (Tolin et al., 2010). The prevalence of CH ranges from 2% to 6% in general community samples of western developed countries, such as Germany and the United States (Iervolino et al., 2009; Timpano et al., 2011). In China, only few studies have investigated CH behaviours (Timpano et al., 2015; Xu et al., 2015), and the prevalence of compulsive hoarding disorder has not been systematically studied. A study with a large-scale representative sample conducted in Germany has shown that approximately two-thirds of participants who have exhibiting CH are also suffering from CB, which suggests a close association between these two compulsive behaviours (Mueller et al., 2009). Hoarding compulsive buyers have reported more severe buying symptoms and obsessive–compulsive symptoms (Mueller et al., 2007). This may be explained by the fact that excessive or maladaptive object attachment is the defining characteristic of both CH and the acquisition process within CB (Moulding et al., 2021). For compulsive hoarders, they develop an excessive attachment to the things they possess and fail to discard, while for the compulsive buyers, they display an uncontrollable desire to own something immediately, which can also be considered is a kind of maladaptive object attachment. Therefore, it is possible that both CH and CB can be displayed by the same individual and the desire to hoard may lead to CB to some extent. Taking these findings together, this study explores the compulsive hoarding behaviours in China and further hypothesizes that CH behaviours are closely related to and predictive of CBB (Hypothesis 3).

This research examines CBB in China using a general community sample in Hong Kong and Mainland China and explores the relationships amongst CBB, CH behaviours, age and gender.

## Methods

### Participants

This epidemiological study employed population-based cross-sectional design. The inclusion criteria were age of 18 years old or older, living in the studied regions, and able to understand Chinese. A sample of 2,305 Chinese participants (based on Cochran formula, Lwanga and Lemeshow, 1991) was planned to recruit from Hong Kong and Shenzhen, a major city of Mainland China.

### Measures

This research constructed a self-reported questionnaire with three sections, i.e., demographics, Richmond Compulsive Buying Scale-Traditional Chinese version (RCBS-TC, He et al., 2018; Lam et al., 2018) and Chinese version of Hoarding Rating Scale (CHRS, Tolin et al., 2010; Liu et al., 2020). RCBS-TC was composed of six items measured with seven-point Likert scale ranging from “1 = totally disagree” to “7 = totally agree.” The total score could range from 7 to 42, wherein the higher the score, the greater the CBB. A sample item was ‘Others may consider me a shopaholic’. RCBS-TC was proven to be well adapted to the Chinese population with a satisfactory internal consistency (Cronbach’s α = 0.88), stability (two-week test-retest intraclass correlation coefficient = 0.82) and two-factor model structure (goodness-of-fit indices: χ^2^/df = 8.56, CFI = 0.99, NFI = 0.98, IFI = 0.99 and RMSEA = 0.09) (Lam et al., 2018). He et al. (2018) re-examined the cut-off value and recommended the value of 29 for determining CB in Chinese population, applicable to Mainland Chinese and Hong Kong (He et al., 2021).

The five-item self-reported CHRS contained items of clutter, difficulty discarding, excessive acquisition, distress and impairment (Liu et al., 2020). Each item was measured on a nine-point scale ranging from 0 (none) to 8 (extreme). The total score could range from 0 to 40, wherein the higher the score, the more severe the hoarding behaviours. Based on the latest adaptation studies (Liu et al., 2020), the reliability of CHRS was satisfactory (Cronbach’s α = 0.86, corrected item-total correlation coefficients = 0.60–0.74 and two-week test retest intraclass correlation coefficient = 0.78). The content (CVI = 0.80–1.00 from six healthcare and social science experts in CB), face validity (100% comprehensibility from 20 public samples), factorial validity (exploratory and confirmatory factor analysis) were all satisfactory. One-factor model of CHRS was identified in Chinese population samples (goodness-of-fit indices: χ^2^/df = 2.26, CFI = 0.99, NFI = 0.99, IFI = 0.99 and RMSEA = 0.049).

Previous study has adopted CHRS and RCBS-TC as measurement scales for self-reported correlational research and concluded that common method bias between compulsive hoarding and buying as measured by self-reported method is not demonstrated in obsessive-compulsive continuum, which hence provided satisfactory evidence that both scales is applicable in Chinese (He et al., 2021). Supplementary Materials 1, 2 provided the items and format of RCBS-TC and CHRS.

Demographic items, such as age, gender, marital status, education level and monthly income, were also recorded for analysis.

### Procedure

Electronic and paper-based data collections were used complementarily. Some participants were recruited from the general public in three districts of Hong Kong, i.e., Kowloon, Hong Kong Island and New Territories, thereby exhibiting a fair mix of people with different backgrounds. Research assistants invited pedestrians to complete self-administrated questionnaires (including demographic questionnaires, RBCS-TC and CHRS). The paper-and-pencil method was also used for the data collection. Participants from Shenzhen were recruited online and completed the online survey voluntarily. Ethical approval was sought from the ethical committee of a local university and the collaborative organisation. Informed consent was obtained from the participants through appropriate method (e.g., verbal consent for the general public participants recruited in railway stations and written consent in the online questionnaire).

## Results

**FIGURE 1:**
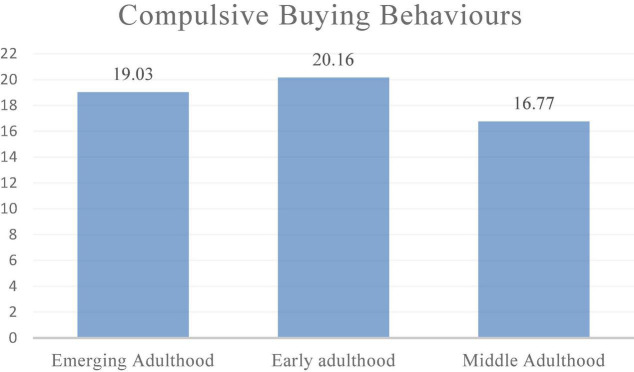
Compulsive Buying Behaviours among three age groups.

A total of 2,439 valid samples (response rate = ∼38%) were collected. With regard to participants’ demographic characteristics, 47.8% (52.2%) were males (females) and their ages ranged from 18 to 59 years. The valid samples were divided into three age groups, i.e., emerging adulthood (ages 18–29 years, *n* = 1,203), early adulthood (ages 30–39 years, *n* = 642) and middle adulthood (ages 40–59, *n* = 594). Table 1 summarized sample demographics and means of compulsive buying. A significant age difference was found across the three age groups (*F* = 30.98, *p* < 0.001, see Figure 1). CBB reached its peak for the early adulthood group (*M*_early_ = 20.16, *SD* = 8.09) and then dropped in the middle adulthood group (*M*_middle_ = 16.81, *SD* = 7.60), which was less severe than the emerging adulthood group (*M*_emerging_ = 19.03, *SD* = 7.39). By using the value of 29 as the cut-off point to separate compulsive from non-compulsive buyers (He et al., 2018; Lam et al., 2018), the prevalence of CBB across the three age groups was 11.3%, 18.5% and 8.1%, respectively. Thus, Hypothesis 1 was partly supported. In particular, the findings showed that younger adults (i.e., emerging and early adulthoods) were more prone to CBB. The screening of hoarding behaviours displayed a similar inverted u-shaped development (*F* = 14.34, *p* < 0.001, *M*_emerging_ = 15.10, *SD* = 7.66; *M*_early_ = 16.04, *SD* = 7.71; *M*_middle_ = 13.68, *SD* = 8.10).

**TABLE 1:** Sample demographics and means of compulsive buying by age group.

		Emerging adulthood		Early adulthood		Middle adulthood	
		N	Mean (SD)	N	Mean (SD)	N	Mean (SD)
Gender	Male	552	16.6(6.92)	295	18.04(7.77)	317	15.28(7.29)
	Female	649	21.09(7.15)	347	21.96(7.93)	277	18.56(7.58)
		*t* = −11.00	*p* < 0.001	*t* = −6.31	*p* < 0.001	*t* = −5.37	*p* < 0.001
Partners	Without	1013	18.62(7.34)	229	18.14(8.06)	125	17.18(7.42)
	With	188	21.23(7.28)	413	21.28(7.89)	469	16.71(7.65)
		*t* = −4.50	*p* < 0.001	*t* = −4.80	*p* < 0.001	*t* = 0.61	*p > 0.05*
Education	Primary school or below	39	19.59(8.60)	17	15.59(8.16)	76	13.38(6.42)
	Secondary school	302	18.68(8.09)	134	18.36(8.26)	205	16.14(7.30)
	Higher education	295	19.19(6.89)	105	21.02(8.26)	89	16.96(7.61)
	Bachelor’s degree	522	19.08(7.09)	312	21.15(7.89)	172	18.92(8.08)
	Master’s degree or above	43	19.14(8.17)	74	19.09(7.45)	52	17.19(6.68)
		*F* = 0.27	*p > 0.05*	*F* = 4.92	*p* < 0.01	*F* = 7.99	*p* < 0.001
Income	< 10000	324	18.30(7.17)	20	20.60(5.92)	33	13.97(6.93)
	10001–20000	345	19.46(7.40)	151	18.76(8.02)	134	16.41(7.43)
	20001–40000	318	19.07(7.43)	228	20.76(7.74)	149	17.30(7.59)
	40001–60000	139	19.71(7.38)	142	19.54(8.37)	139	16.83(7.73)
	> 60001	68	18.31(7.95)	61	20.05(8.25)	94	17.01(7.20)
		*F* = 1.55	*p > 0.05*	*F* = 1.57	*p > 0.05*	*F* = 1.44	*p > 0.05*

Females demonstrated more compulsive buying behaviours than males in all three age groups (See Table 1). Thus, Hypothesis 2 is supported. The participants with partner showed significantly less CBB than those without partner in their emerging and early adulthood (*t*_emerging_ = −4.50, *p* < 0.001; *t*_early_ = −4.80, *p* < 0.001). However, the differences were not observed in the middle adulthood (*t*_*middle*_ = 0.61, *p* > 0.05). As shown in Table 1, the participants with different education levels showed significantly different tendencies of CBB in both early and middle adulthood (*F*_early_ = 4.92, *p* < 0.01; *F*_middle_ = 7.99, *p* < 0.001), whereas not in the emerging adulthood (*F*_*emerging*_ = 0.27, *p* > 0.05). Across three age groups, there was no significant effect of income on CBB.

Compulsive buying (CB) was found significantly and positively correlated with CH in all three age groups (*r*_*emerging*_ = 0.47, *r*_*early*_ = 0.53, *r*_*middle*_ = 0.47). To investigate further the salient predictors for CBB in each age group, binary logistic regressions were conducted with the probability to be a compulsive buyer as dependent measurement and compulsive hoarding and demographic variables as predictors. Because of the insignificant effect of income found across the three age groups, income was not included in the binary logistic regressions. As shown in Table 2, in the emerging adulthood, significate effects of CH, gender, and partner were observed. However, education level and partner, the variables originally affected CBB in early adulthood were found insignificant in predicting CBB while the effects of CH and gender remained significant. As for the middle adulthood, only CH and education level were the significant predictors of CBB, which is contradicted to the findings reported above where gender difference was found significantly in CBB for this age group. In summary, Hypothesis 3 was supported.

**TABLE 2:** Binary logistical regression predicting compulsive buying.

Age group		B	S.E.	Wald	df	Sig.	Exp(B)	95% CI	
								Lower	Upper
Emerging adulthood	Hoarding	0.126	0.015	73.110	1	0.000	1.135	0.098	0.157
	Gender	1.066	0.227	22.096	1	0.000	2.905	0.673	1.537
	Partner	0.557	0.233	5.708	1	0.017	1.745	0.047	1.006
	Education	–0.131	–0.104	1.582	1	0.209	0.877	–0.372	0.096
	Constant	–4.715	–0.469	101.099	1	0.000	0.009	–5.858	–3.749
Early adulthood	Hoarding	0.143	0.017	69.313	1	0.000	1.154	0.110	0.186
	Gender	0.806	0.243	10.970	1	0.001	2.239	0.343	1.292
	Partner	0.420	0.257	2.675	1	0.102	1.522	–0.097	0.939
	Edu	0.163	0.120	1.853	1	0.173	1.177	–0.097	0.435
	Constant	–5.496	0.622	78.094	1	0.000	0.004	–7.049	–4.331
Middle adulthood	Hoarding	0.130	0.022	34.949	1	0.000	1.139	0.082	0.190
	Gender	0.455	0.327	1.935	1	0.164	1.577	–0.147	1.182
	Partner	–0.291	0.401	0.528	1	0.468	0.747	–1.000	0.586
	Edu	0.301	0.140	4.626	1	0.031	1.351	0.066	0.546
	Constant	–5.571	0.722	59.524	1	0.000	0.004	–7.457	–4.275

## Discussion and Conclusion

This research aimed to provide a clear picture of the prevalence of CB and CH in a general Chinese sample from emerging to middle adulthood. CB and CH behaviours displayed an inverted u-shaped pattern across three different age groups. This finding partially supported Hypothesis 1 and showed that younger adults were strongly affected by CBB compared with older adults. The prevalence of CBB across the three age groups was 11.3%, 18.5%, and 8.1%, respectively. In addition, the strength of correlation between the two compulsive behaviours was consistent and maintained across the three age groups. These results suggested that CB may continually increase (or perhaps develop) in the emerging adulthood, reach the peak at the early adulthood and drop slightly in the middle adulthood in these two major cities of China. This newly discovered pattern of compulsive disorders in Chinese population was quite different from the findings based on American and European population. By studying the data obtained from the United Kingdom, Dittmar (2005b) found that age and CBB were significantly and negatively correlated and European participants under 35 years old scored much higher than those over 35 years old. However, in this study, the participants in the early adulthood displayed more CBB than their younger and older counterparts. These differences could be due to cultural differences among these societies. In Chinese societies, Confucianism exerted great impact on individuals’ thoughts and behaviours. Confucius once said, “since 30, I have been well established (i.e., *San shi er li*, “三十而立”).” Thus, Chinese people in their thirties were expected to be accomplished and have acquired certain social status. Meanwhile, the unique concept of face (i.e., *Mianzi*) in Chinese culture could be used to explain the high prevalence of CB amongst online buyers who were found to shop more often to show off their social statuses and success (He et al., 2018). Therefore, early adulthood individuals who have already been working for several years after their college graduation were more inclined to exhibit excessive shopping behaviours and own a large number of possessions to maintain and enhance their social status. By contrast, the younger adults were free from the strong urge to exhibit such behaviours, given that they were still young and studying in universities. Thus, future studies could explore whether individuals in the early adulthood demonstrate higher rate of observed buying than unobserved ones in comparison with other age groups to examine further the impact of culture on individuals’ CB and CH behaviours. However, the overall prevalence of CBB in the middle adulthood in the current study is significantly lower compared with the other two younger groups, which may be explained by the cognitive maturity of men and women in this age group. As they enter their 40s and 50s, their urge to show off their social statuses and success may decrease as mature adults and they may also learn to use a better way to cope with their negative feelings, which could lead to fewer compulsive buying and hoarding behaviours.

Consistent with the findings of studies conducted in Western societies and as predicted in Hypothesis 2, Chinese females with ages between 18 and 39 years were more prone to CB and CH behaviours, which is consistent with the past findings. Both Chinese and Western studies suggested that young female compulsive buyers may use excessive shopping to cope with their stress and negative feelings (Challet-Bouju et al., 2020; Zheng et al., 2020). However, as people grow older, both genders seemed to be influenced by these compulsive behaviours equally as gender was considered insignificant predictor of CBB in the middle adulthood. The gender differences observed in CBB in this study could be at least partially explained by the traditional gender roles in a Chinese family. In a traditional Chinese family, females were expected to take care of the whole family and perform all the grocery shopping for the family, which could lead to females spending more time in supermarkets and shopping malls (Sun, 2013). Therefore, shopping for Chinese females could be perceived as an enjoyable leisure activity and part of their expected responsibilities to their families. By contrast, Chinese males were shaped as breadwinners who support the family financially, which could decrease their inclination in shopping and spending money, especially in their emerging and early adulthood when they should be striving for their career success and busy earning money for their future family (Sun, 2013). However, when Chinese males enter into their middle or older adulthood, their careers were already on the right track and they had succeeded in fulfilling social obligations to their family. Thus, Chinese males could start to satisfy their own needs and wants, which would lead to compensatory buying or CB. Chinese males could also exhibit such behaviours to deal with the negative emotions they developed from their stressful work and failure in supporting their family. In this case, shopping and hoarding could offer a way out. For instance, a study investigating Italian elderly men’s shopping behaviours showed that approximately 30% of the elderly men were affected by CBB and exhibited problematic shopping behaviours as a means to deal with their supressed feelings (Varveri et al., 2014). These findings could explain why the gender differences in CBB disappear when individuals enter into their middle or older adulthood.

The association between CB and CH was proven in this research (supported Hypothesis 3), which was consistent with existing literature. In a study conducted in Germany, two-thirds of the participants who were suffering from compulsive hoarding were also classified as compulsive buyers (Mueller et al., 2009). Similar findings were also found in studies with American samples (Frost et al., 2002). Thus, scholars argued that compulsive buyers could share similar beliefs of possession and buying as compulsive hoarders (Kyrios et al., 2004). Compulsive hoarding behaviours were found to be a robust predictor of CBB across the three age groups, which indicated that compulsive buyers shop, at least to some extent, because of their needs of possession and hoarding. This is in line with the past literature that both CH and the acquisition process withing CB involve individuals’ excessive or maladaptive object attachment (Moulding et al., 2021). However, a study in Taiwan suggested the other way around and their data showed that Taiwanese compulsive buyers were driven by an obsessive need to acquire products rather than the need to collect and hoard these products (Lo and Harvey, 2014). However, the sample size in the above study was relatively small with less than 200 participants. To further elaborate existing understanding of the relationship between CB and CH, more research with larger sample size and representative population should be conducted in future.

The findings of this study could potentially have important society and treatment implications. The inverted u-shaped pattern across the three age groups of CB and CH suggested that society should care about the mental health of individuals in the early adulthood. Establishing identity, one of the developmental tasks of young adults (Havighurst, 1972), together with the influence of Confucian teachings (i.e., “since 30, I have been well established”) may exert impact on the Chinese CB and CH behaviours. CB and CH were also considered identity substitutes and associated with materialistic value endorsement and depression (Claes et al., 2016). Mass media should reduce their emphasis on materialistic values to prevent further increase in the prevalence of CB and CH. From the findings of the binary logistic regression, gender differences were only found in the participants of the emerging and early adulthood groups, but not in the middle adulthood group. The disappearance of gender differences in CB and CH in this age group showed that both male and female middle adulthood consumers could benefit from “retail therapy.” On the other hand, these findings also serve as a signal that the helping professionals in the field of mental health should pay more attention to the middle-aged males’ buying behaviours, which previously was seldom the focus of CBB, to identify their problematic coping with the stress and negative feelings. Besides, the association between CB and CH found in the current research showed that the clinical experts should think over the comorbidity of the two psychological disorders.

Despite of significant contributions of this study, some limitations should be observed. Firstly, we adopted two well validated scales for screening two compulsive behaviours. However, precluding response bias was difficult due to the nature of self-reported answers. Secondly, due to the limitation of funding, we were unable to recruit psychiatrists to perform the diagnosis of CB and CH that was regarded as gold standard for all 2,439 participants. Such shortcomings could affect the accuracy of categorisation of compulsive and non-compulsive group. Lastly, this study recruited large-scale participants to represent Chinese general population. However, the age distribution was uneven. The number of the participants composing the early and middle adulthood was half of that of the emerging adulthood.

To the best of our knowledge, this study was one of the pioneers to explore the prevalence of CB and CH behaviours in a Chinese community sample. The prevalence of these compulsive behaviours was displayed in an inverted u-shaped pattern, which was different from those of western societies. Demographic variables, such as gender, marital status, income and education level, were found to affect these compulsive behaviours, especially in the emerging and early adulthood. These newly discovered Chinese findings could be explained by the unique Chinese culture. Thus, future research should further explore how culture influences existing understanding of CBB.

## Data Availability Statement

The raw data supporting the conclusions of this article will be made available by the authors, without undue reservation.

## Ethics Statement

The studies involving human participants were reviewed and approved by Ethical Review Regarding Human Research, The Open University of Hong Kong (Ref no. HE20Jul2017-S&T2017/01). The patients/participants provided their written informed consent to participate in this study.

## Author Contributions

SL and HH: conceptualization and funding acquisition. JY, SL, and HH: methodology and writing – review and editing. JY and SL: writing – original draft preparation. SL: project administration. All authors contributed to the article and approved the submitted version.

## Conflict of Interest

The authors declare that this study received funding from three parties, including The Open University of Hong Kong, Wofoo Social Enterprises, Hong Kong, and National Natural Science Foundation of China. The funder was not involved in the study design, collection, analysis, interpretation of data, the writing of this article or the decision to submit it for publication.

## Publisher’s Note

All claims expressed in this article are solely those of the authors and do not necessarily represent those of their affiliated organizations, or those of the publisher, the editors and the reviewers. Any product that may be evaluated in this article, or claim that may be made by its manufacturer, is not guaranteed or endorsed by the publisher.
