# Supplementary material for: The Prevalence of Compulsive Buying and Hoarding Behaviours in Emerging, Early, and Middle Adulthood: Multicentre Epidemiological Analysis of Non-clinical Chinese Samples
Source: Front Psychol. 2021 Dec 9;12:568041. doi: 10.3389/fpsyg.2021.568041 (PMC8696278; doi:10.3389/fpsyg.2021.568041)
Supplement: Supplementary file 1 [file Table_1.DOC]

**Supplementary Material 1**

**里奇蒙強迫性購物量表－繁體中文版**

**Richmond Compulsive Buying Scale- Traditional Chinese Version (RBCS-TC)**

| **購物習慣** | |
| --- | --- |
| 請您針對每題所敘述的內容，選出您認為與實際情況符合的程度。 | |
|  | 非常　　　　　　　　　　　　　　　　　非常  不同意 ＜──────────────────＞ 同意 |
| 1. 我的櫃裡有未開包裝的物品。 | １　　２　　３　　４　　５　　６　　７ |
| 1. 其他人可能認為我是購物狂。 | １　　２　　３　　４　　５　　６　　７ |
| 1. 購物幾乎是我生命的全部。 | １　　２　　３　　４　　５　　６　　７ |
| 1. 我認為我是個衝動的消費者。 | １　　２　　３　　４　　５　　６　　７ |
|  |  |
|  | 從不 ＜───────────────────＞ 經常 |
| 1. 我會買我不需要的東西。 | １　　２　　３　　４　　５　　６　　７ |
| 1. 我會買我未計劃要買的東西。 | １　　２　　３　　４　　５　　６　　７ |

Source of RCBS: Ridgway et al. (2008), Journal of Consumer Research; He et al. (2018), Journal of Business Research.

Source of RCBS-TC: Lam et al. (2018), Journal of Behavioral Addictions.
